# OTA 33-B1 intra-articular fracture of the distal femur of the lateral femoral condyle involving both weight-bearing and non-weight-bearing surfaces after acute patellar dislocation: a case report and literature review

**DOI:** 10.3389/fsurg.2026.1835416

**Published:** 2026-10-08

**Authors:** Wanqiong Zhang, Mingjin Li, Xi Liu, Huixu Ma

**Affiliations:** 1 Department of Rehabilitation Medicine, Chongqing General Hospital, School of Medicine, Chongqing University, Chongqing, China; 2 Department of Trauma Orthopaedics, Chongqing General Hospital, Chongqing University, Chongqing, China; 3 Department of Radiology, Chongqing Hospital of Traditional Chinese Medicine, Chongqing, China; 4 Department of Rehabilitation Medicine, Chongqing Academy of Medical Sciences, Chongqing General Hospital, School of Medicine, Chongqing University, Chongqing, China

**Keywords:** medial patellofemoral ligament, open reduction and internal fixation, OTA 33-B1 intra-articular distal femoral fracture, patellar dislocation, weight-bearing

## Abstract

**Background:**

Concomitant patellar dislocation with OTA 33-B1 intra-articular fracture of the distal femur involving both weightbearing and non-weightbearing lateral femoral condyle fracture is a rare entity, with few case reports in the literature, and no standard approach to its treatment. In this study we review previous cases along with describing one patient presenting to our institution.

**Methods:**

A systematic literature search in PubMed/MEDLINE and Web of Science was conducted using the key words “patella dislocation,” "medial patellofemoral ligament injury”, “OTA 33-B1 lateral femoral condyle fracture” to retrieve related articles. Data extracted included patients’ demographics (age, sex),injury mechanism, type of dislocation, location of fracture, treatment modality, and outcome. In addition, a detailed description of the course of this patient's disease is presented and discussed.

**Results:**

This study included 7 patients including reported literature in previous publications as well as one primary case from our own institute. The mean age of the patients was 19 years, with most fractures involving the weight bearing area of the lateral femoral condyle(OTA 33-B1).Based on the symptoms compared, injury mechanism, management modality and outcome. This study provides more information on this rare type of fractures from a clinical point of view which may be useful in diagnosis as well as treatment planning and further research studies.

**Conclusions:**

Injuries to the lateral femoral condyle(OTA 33-B1 intra-articular fracture of the distal femur), involving both weightbearing and non-weightbearing portions are extremely uncommon sequelae of a patellar dislocation. The mechanism of injury is probably due to direct impaction of the patella with additional shear stress imparted by the tibial plateau. Resulting in comminuted fragmentation of the bones in these articulations.

## Introduction

The health consequences associated with acute patellar dislocation are potentially serious because of the high recurrence rate and potential sequelae. The sequelae include chondral injury of the patellofemoral compartment, bone or cartilage breakage (OCF), damage to the inner soft tissue, chronic pain, limited range of motion and finally osteoarthritis within the patellofemoral joint (1). The most common complications include OCF which has been reported to occur in up to 58% or as low as 31% of patients after patella dislocations acutely. In general, OCF are caused by an impact on the lateral femoral condyle due to a collision between the medial aspect of the patella and lateral distal femoral epicondyle: either during the dislocation episode or while the patella is being relocated (2). The two regions that have been reported to be most often involved with OCF following patella dislocation include the lateral femoral condyle sulcus and medial patella (3). These fractures usually involve certain non-weight-bearing zones, defined as the far lateral marginal area of the lateral femoral condyle outside the main load-bearing trochlear-condylar contact region, including the lateral margin of the femoral sulcus or the lateral border thereof, and which result from a direct blow force. Weight-bearing zones refer to the central main articular surface of the lateral femoral condyle that bears physiological axial load during knee weight-bearing activity. Non-weight-bearing marginal injuries are those confined to the peripheral rim without involvement of the central load-bearing area, which are mostly chondral injuries located marginally along the far lateral aspect of the lateral femoral condyle, proximal to the sulcus terminalis, or both. The mechanism (4) is different in the case of OTA 33-B1 intra-articular fracture of the distal femur involving the load-bearing part of the lateral femoral condyle, which are more rare. This kind of trauma is associated with dislocations of the patella occurring during knee flexion positions (5). Although in a single case report, there were two instances where an isolated OCF was observed at the weight-bearing region on the lateral side of the femoral condyle (6), comminuted OTA 33-B1 intra-articular fracture of the distal femur in both bearing and nonbearing portions of the lateral femoral condyle after patella dislocation has only been described sparsely in the scientific literature (7). In this report we describe a unique presentation of a comminuted OTA 33-B1 intra-articular fracture of the distal femur involving both the weight-bearing and non-weight-bearing zones of the lateral femoral condyle following acute patellar dislocation. It analyzes the potential injury mechanism and key clinical features of this unusual injury pattern, and summarizes the clinical characteristics and treatment strategies for acute patellar dislocation combined with lateral femoral condyle fracture through a systematic review of previously published cases.

## Methods

A systematic literature search was conducted in PubMed, Embase and Web of Science to identify published clinical cases related to OTA 33-B1 intra-articular fracture of the distal femur following acute patellar dislocation, covering all studies published up to March 2026.The search terms included natural language and controlled vocabulary words related to patellar dislocation, patella lateral retinacular ligaments, patella medial stabilising ligaments, patellar tendons, cartilage bone injury, and lateral femoral condyle fracture.

We also handsearched references of retrieved articles for additional relevant studies. We selected those reports where a clinical case or series was reported with information regarding management of OTA 33-B1 intra-articular fracture of the distal femur after acute patellar dislocation, excluding all articles related to osteoarthritis occurring in elderly patients, non-trauma cases or lack of enough details regarding surgery. The result is presented in Table 1. All this information was collected, including the authors, year of publication etc., patient characteristics (age, gender), cause of the injury, bone parameters (site, dimension of OTA 33-B1 fracture), imaging features, surgery type and post-operative recovery regimen, the frequency of outpatient review, and the clinical as well as the functional outcome.

**Table 1:** Literature review of previously reported cases of OTA 33-B1 intra-articular fractures of the distal femur involving the lateral femoral condyle following acute patellar dislocation

Author (year)	Age/gender	Mechanism	Dislocation Type	Fracture Location	Clinical Symptoms	Imaging Findings	Treatment Method	Postoperative Management	Follow-up Time	Outcome and Prognosis
Chan et al. (2014) (3)	12 years/M	Torsion injury (with history of patellar dislocation)	Lateral patellar dislocation	Pure cartilage fracture in the weight-bearing area of the lateral femoral condyle (4 × 2.2 cm)	Massive knee effusion, tenderness in the medial patella and distal lateral femur, and restricted mobility	x-ray (-); MRI: extensive cartilage delamination at lateral femoral condyle weight-bearing area, medial patellar cartilage fragment displacement	Lateral parapatellar incision: cartilage fragment reduction + absorbable suture anchor (Vicryl suture) + fixation with 5 polylactic acid absorbable nails (SmartNail); concurrent MPFL repair	Hinge brace, 0 -30 range of motion, increasing by 15° per week; non-weight-bearing for 6 weeks, full weight-bearing at 8 weeks.	11.5 months (secondary exploration due to instability during MPFL reconstruction)	Cartilage plate healed stably with near-normal morphology; no knee pain, but second-stage MPFL reconstruction performed for patellar instability.
Cavalheiro et al. (2018) (4)	13 years/M	Falling during football	Lateral patellar dislocation	A chondrocartilaginous fracture (3.0 × 1.5 × 0.3 cm) primarily involving the cartilage in the weight-bearing area of the lateral femoral condyle	Swelling of the knee joint, severe pain, limited mobility (20–110), and inability to fully extend the joint	x-ray/CT: intra-articular free bone fragments (lateral femoral condyle weight-bearing area), trochlear dysplasia, patellar tilt	Phase I: Lateral parapatellar approach, cartilage fragment reduction + suture through bone (PDS suture) + fibrin glue. Phase II (9 months later): Reconstruction of MPFL and MPTL (thinnest femoral tendon)	1st phase: knee extension brace fixation for 4 weeks, then non-weight-bearing immobilization for 6 weeks; arthroscopic release at 3 months for joint adhesion.	16 months (since the first surgery)	Painless, ROM 0 - 140 (pre-injury mobility restored); MRI: intact cartilage continuity, markedly improved functional scores
Callewier et al. (2009) (7)	23 years/M	Martial arts: soft leg strike (knee extension force	Lateral patellar dislocation	Osteochondral fracture in the weight-bearing area of the lateral femoral condyle (3 × 1 cm)	Knee joint swelling, pain, limited mobility, 10° flexion contracture	x-ray: intra-articular free fragments; CT: large osteochondral fracture, anteroinferior femoral condyle	Open reduction with 5 polydioxanone absorbable screws fixation via the lateral parapatellar approach	The knee brace was fixed for 6 weeks, and weight-bearing was prohibited for 3 months.	12months	Painless procedure, ROM 0 -130, sports resumption; MRI: good fracture healing, implant absorption
Delniotis & Leidinger (2024) (8)	13 years/M	generation) Falling (bathroom)	Lateral patellar dislocation	Pure cartilage fracture of the lateral femoral condyle (2.4 × 1.35 cm^2^)	Knee joint effusion with lateral pain, no abnormalities observed on initial x-ray	weight-bearing area US: floating cartilage fragments; MRI: cartilage fracture, MPFL tear, trochlear dysplasia	Midline incision + cartilage flap reduction + suture bridge technique fixation (PDS sutures + bone tunnel)	6 weeks weight-free, 2 weeks partial weight-bearing; 2 weeks straight fixation, 2–4 weeks knee flexion ≤60,4 -6 weeks ≤90°	4 months	MRI shows good healing, with 25° flexion limitation and painless walking
Bolisetti et al. (2025) (9)	22 years/F	occurs upon standing up after a knee injury	Lateral patellar dislocation	Posterolateral femoral condyle osteochondral fracture fragment embedded in medial patella, with a large medial patellar osteochondral defect.	Knee swelling, pain, tenderness over medial joint line and limited ROM (0 -80)	CT/MRI: Femoral condyle osteochondral fragment (medial patella), large medial free fragment	Arth + open: femoral condyle fragment reimplantation fixation (K-wire+4 SmartNails), medial patellar fragment fixation, retinaculum release.	Weight-bearing was permitted immediately after surgery, with the use of a hinged brace to lock the extended position.	8 months	Pain resolved, full range of motion restored, ligament stability achieved, no re-dislocation
Shah et al. (2025) (11)	20 years/M	Basketball injury	Lateral patellar dislocation (self-reduction)	Posterior-lateral fracture of the lateral femoral condyle (2.5 × 2.5 cm)	Swelling, pain, and limited mobility (0-40) of the knee joint with lateral instability	x-ray, CT and MRI showed a large osteochondral fragment, complete MPFL rupture, patellar alta and increased TT-TG distance.	Arthroscopy + lateral parapatellar reduction + absorbable/headless screw fixation + MPFL reconstruction (semitendinosus/gracilis autograft)	Long leg brace, no weight-bearing for 4 weeks, partial weight-bearing after 4 weeks, early range of motion training	6 months	Fracture healed with range of motion 0 - 120, no re-dislocation, and can resume ball games

## Results

### Case series overview

After systematic retrieval and screening, we included 6 articles; together with the index case, this series included 7 patients (6 cases in Table 1 plus 1 case from our institution). The average age at initial diagnosis was 19 years (SD 6.11; range 12–30 years), suggesting that the majority of patients were adolescents. Females accounted for 14% (1/7), and males accounted for 86% (6/7). The patients in this cohort were all diagnosed with a lateralized patella and the majority had a history of trauma from an indirect mechanism (e.g., sports).fallen down accidentally; and twisting. Most common site for fractures was posterior articular facet of the lateral femoral condyle consistent with OTA 33-B1 intra-articular fracture of the distal femur. Surgical treatments were mainly performed using either an open reduction or arthroscopically assisted reduction and internal stabilization, using different fixation techniques (e.g., bioabsorbable implant, permanent screw, suture anchor, suture bridge construct). Most studies involved simultaneous medial patellofemoral ligament (MPFL) repair or reconstruction. One unique aspect about this case was that there were both weightbearing and non-weight bearing fracture sites of the OTA 33-B1 fracture. After surgery, all patients had the same postoperative management including brace treatment and progressive physiotherapy regimen, and phased weightbearing rehab protocols. Maximum time for clinical follow up was 62 months. All patients showed radiologically healed fractures and considerable improvement of the function of the knee joint:near-complete elimination of the patient's pain syndrome, and good therapeutic results. All patients resumed their routine activities and sports practice. One patient out of the seven included in this study had a secondary procedure due to recurrent patellar dislocation (14%).

### Detailed description of the index case

A 30-year-old male presented to the ER with pain and swelling in the left knee for 2 h following a traumatic injury. The patient has no past history of any related disease process or surgery in the same area. Physical examination showed extensive bruises to the skin and soft tissue structures in the anterior medial aspect and inferior patellar area of the left extremity. The involved joint had a fixed flexion angle of about 60°, obvious edoema in the joint cavity, normal sensation and pulse in the lower limb.

The emergency radiology was performed immediately, in which computed tomography (CT) showed a large OTA 33-B1 intra-articular fracture of the distal femur involving the weightbearing and non-weight bearing surfaces of the lateral femoral condyle of the injured left knee, and with evidence of recent lateral patellar dislocation by imaging: MRI showed that the MPFL was completely ruptured; an osteochondral flap was displaced and there were multiple chondral separations on both the central loading zone as well as the non-loading zone of the lateral femoral condyle. The osteochondral flap is located medial to the patella (Figure 1). To determine whether any predisposing anatomical factors contributed to the patellar dislocation, preoperative alignment and patellofemoral morphometric parameters were systematically assessed using weight-bearing full-length lower extremity radiographs and axial MRI. Trochlear morphology was first evaluated: the trochlear groove was well-formed, with a sulcus angle of 135° (normal: <145°), and no signs of trochlear dysplasia (Dejour type A–D) were present—there was no crossing sign, no supratrochlear spur, and no double-contour sign. The hip-knee-ankle (HKA) angle was 0°, indicating strictly neutral mechanical alignment (normal: 0° ± 3°), and the mechanical axis passed through the centre of the knee, ruling out valgus malalignment. The tibial tubercle–trochlear groove (TT–TG) distance was 10 mm (pathological threshold: >20 mm), and the tibial tubercle–posterior cruciate ligament (TT–PCL) distance was 21 mm (pathological threshold: >24 mm), both well within normal limits. Patellar height was assessed using the Caton–Deschamps index, which measured 1.0 (normal range: 0.6–1.2), and the Insall–Salvati ratio, which was 1.0 (normal range: 0.8–1.2), confirming the absence of patella alta. Collectively, these measurements demonstrate that no pre-existing anatomical risk factors—specifically trochlear dysplasia, valgus malalignment, elevated TT–TG/TT–PCL distance, or patella alta. After appropriate physical examination and radiographic assessment, we diagnosed this as a partial chondral and subchondral component of the OTA 33-B1 intra-articular fracture of the distal femur of the lateral femoral condyle involving the two articular facets following an acute lateral dislocation of the patella.

**Figure 1:**
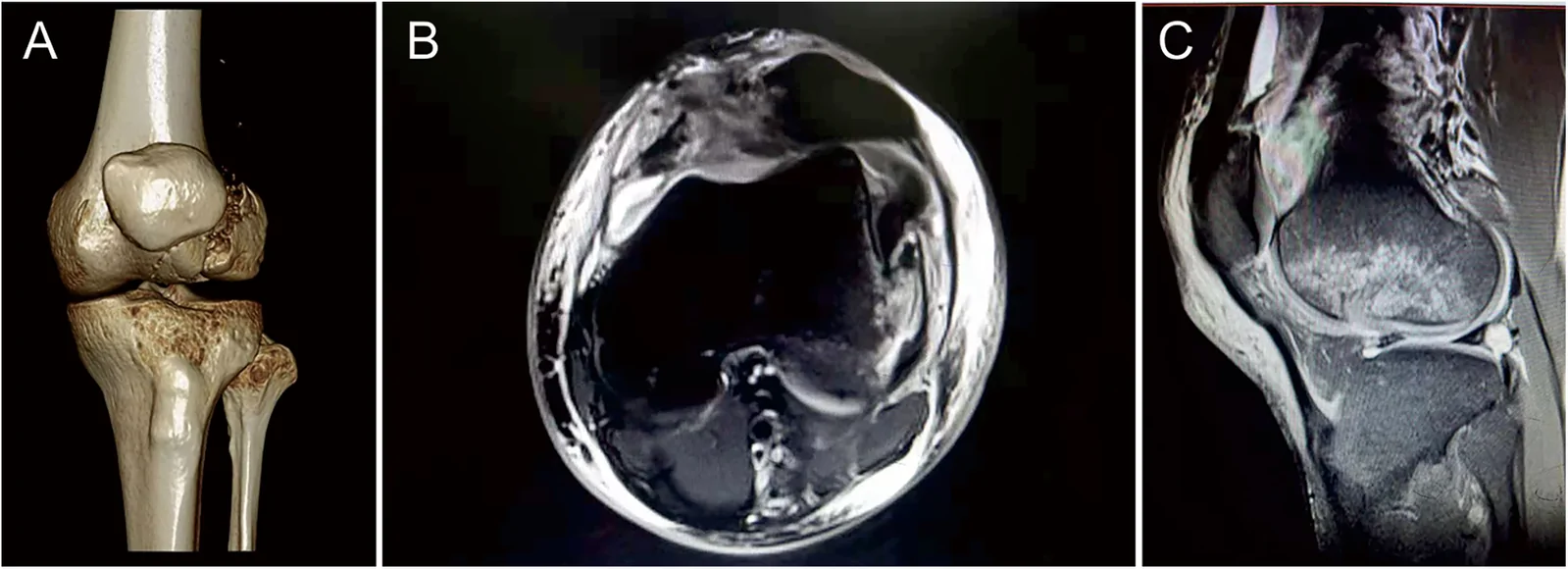
Preoperative imaging of the lateral femoral condyle showing voluminous OTA 33-B1 intra-articular fracture of the distal femur in weight-bearing/non-weight-bearing areas, medial patellofemoral ligament (MPFL) disruption and large chondral delamination. **(A)** CT scan; **(B)** MRI (MPFL view); **(C)** MRI (chondral view).

Because of extensive damage to the load-bearing articular cartilage of the OTA 33-B1 fracture, surgery became a necessity (3). The patient received definitive clinical and radiological diagnosis in the emergency department immediately after trauma. No formal closed manual reduction of the patella was performed preoperatively, as imaging findings indicated spontaneous relocation of the patella after acute lateral dislocation, with persistent soft-tissue instability and articular malalignment remaining. Before the operation, Doppler ultrasonography was used to exclude DVT. Surgery was performed on day seven post-injury with the patient in general anaesthesia, after sufficient resolution of peri-articular soft-tissue swelling. Anterior midline incision was initially attempted (8). During surgery, it was found that MPFL as well as other medial soft tissues were completely and severely torn, besides chondral injury at the medial margin of the patella. For adequate management of the lateral femoral condyle OTA 33-B1 fracture, the operative team made an incision via a medial parapatellar approach, and extended it (9). During surgery, additional evaluation confirmed multiple bony-cartilaginous bodies arising from the non-weight-bearing zone as well as low weight-bearing zones of the lateral femoral condyle related to the OTA 33-B1 fracture were present (7). Anatomical reconstruction was achieved by fixation with parallelly placed lateral to medial running compression screws. Half-threaded screws with a diameter of 3.5 mm were used, and all screw tips were positioned in subchondral bone without penetrating the articular cartilage surface.

Surgical treatment consisted, intraoperatively (Figure 2) (10), of a meticulous reconstruction and reinforcement of the medial patellofemoral ligament. Patellar tracking was assessed intraoperatively, which revealed persistent maltracking with knee flexion due to tight lateral retinacular structures, and indicated a high risk of recurrent patellar dislocation. Accordingly, lateral retinaculum release (LRR) was performed as a secondary adjunctive procedure to realign the patella within the femoral trochlear groove and reduce redislocation risk (9).

**Figure 2:**
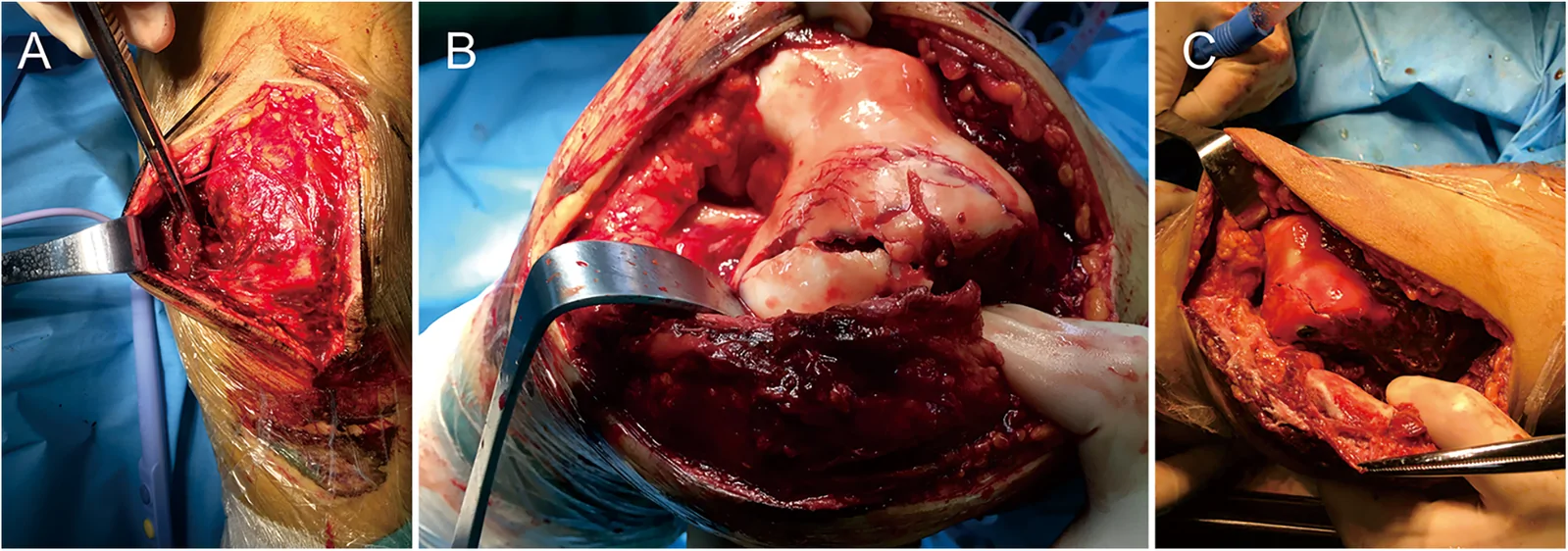
**(A)** Severe medial patellofemoral ligament (MPFL) injury with surrounding soft tissues. **(B)** Fracture fragments on the lateral femoral condyle area related to the OTA 33-B1 intra-articular fracture of the distal femur. **(C)** The femoral condyles are fixed by inserting the cannulated screws from laterally to medially.

Postoperative radiographs confirmed anatomical reduction of the OTA 33-B1 fracture with stable fixation and restored congruity of the lateral femoral condyle (Figure 3). The patient received standard postoperative treatment according to our protocols. For prophylaxis of DVTs, we used LMWH beginning from 8 h after operation (11). The operated leg was immobilised with a hinged orthosis at the knee joint. For the first two weeks postoperatively, the orthosis was locked to allow an arc of motion from full extension to 30° of flexion. Thereafter, the allowed flexion range was progressively increased by approximately 15° per week, with the goal of achieving 90° of flexion by six weeks postoperatively and unrestricted active range of motion thereafter. We kept the patient in full non-weight bearing status for six weeks and started with partial weight bearing as soon as the patient could reach 90° of knee flexion. At three months postoperatively, the patient had reached full weight bearing status. At 19 months post-op, surgical hardware removal was planned, showing residual small osteochondral defect related to the original OTA 33-B1 fracture in the site of original injury (10).

**Figure 3:**
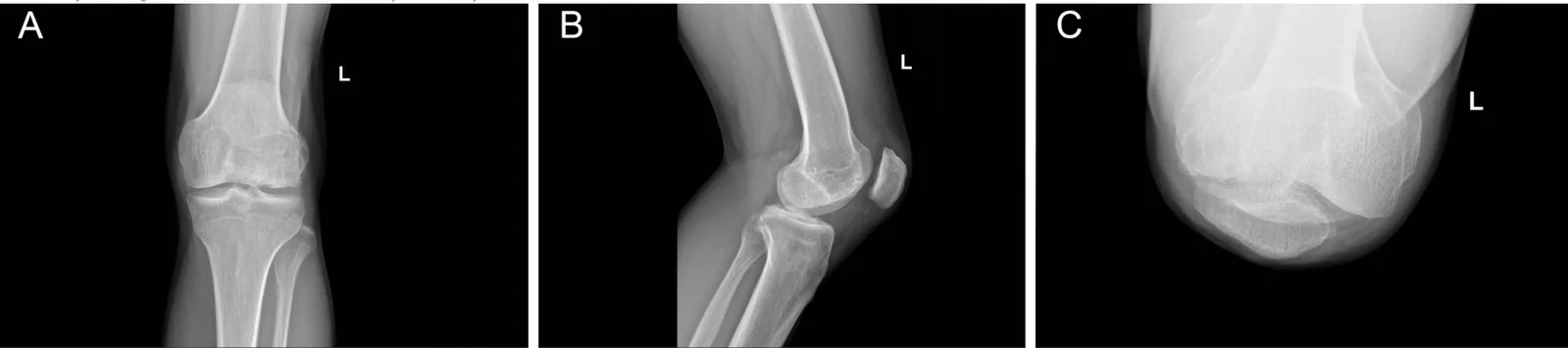
**(A)** Anteroposterior radiograph shows complete fracture healing of the femoral condyle with obliteration of previous screw track lucencies. **(B)** Lateral radiograph confirms solid fracture union of the femoral condyle with resolution of implant-related radiolucency.**(C)** Axial view reveals well-maintained patellar tracking within the trochlear groove, without evidence of recurrent patellar subluxation or dislocation.

At long-term follow-up (at 62 months), this patient showed excellent outcome: reported complete pain relief, and reported a very good or excellent satisfaction level. Objective physical examination revealed unremarkable gait, knee full extension, range of motion (ROM) between 0 and 120°, no patellar maltracking and articular step-off. Outcome showed that no restriction was experienced either with climbing stairs or undertaking mild exercise, which were undertaken pain free. A follow-up MRI scan revealed a well integrated medial patellofemoral ligament reconstruction with no evidence of patellar dislocation, whereas there were still traces of the initial osteochondral break of the OTA 33-B1 fracture on the weightbearing part of the round prominence of the outside femur (Figure 4) (9).

**Figure 4:**
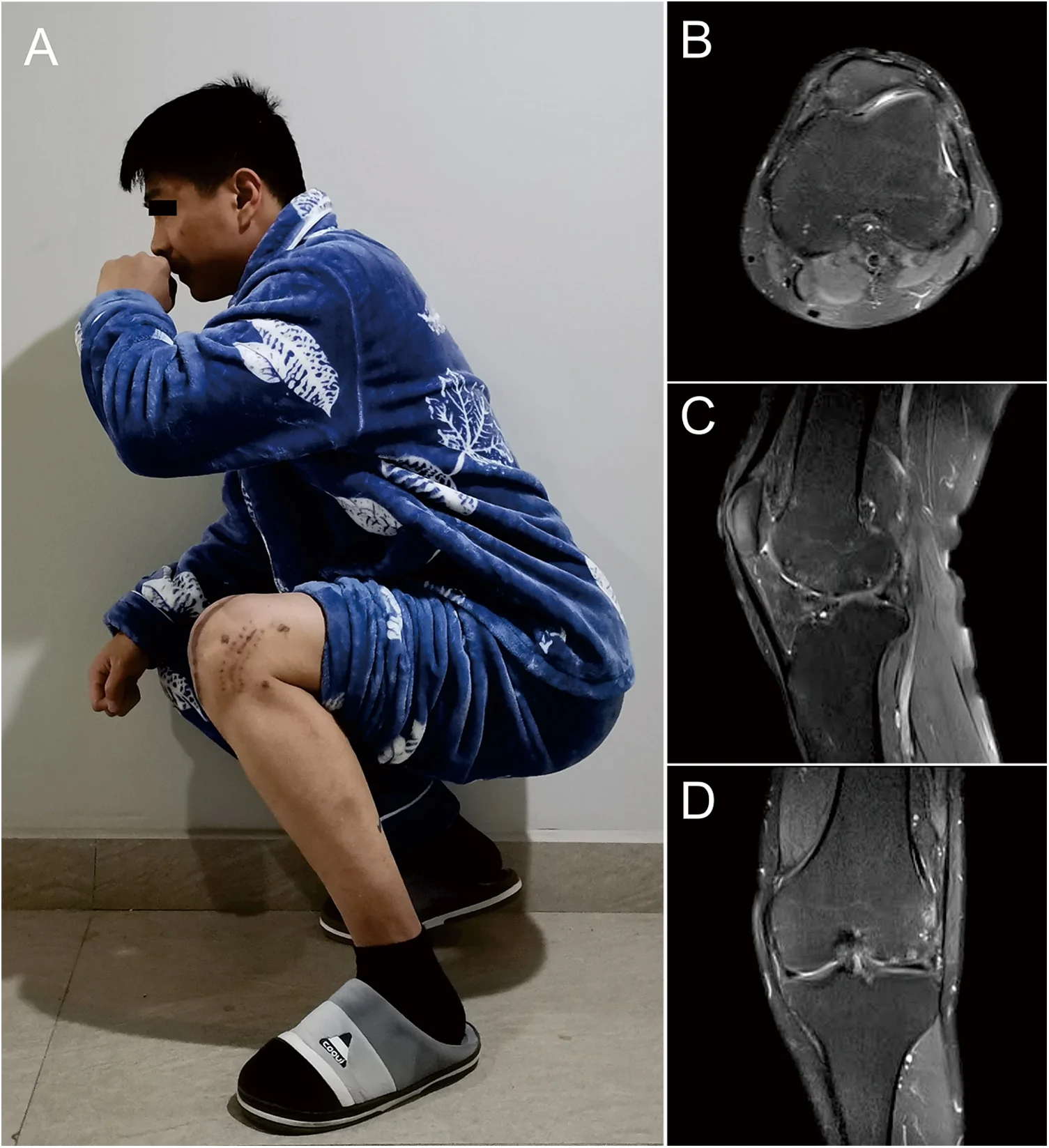
**(A)** Clinical photograph demonstrating extension to 120° at the knee joint; **(B–D)**. Subsequent MR images. **(B)** Cartilage damage within the weight-bearing region on the lateral side of the femoral condyle related to the OTA 33-B1 intra-articular fracture of the distal femur; **(C,D)** Complete reconstruction of MPFL with no signs of instability.

## Discussion

It has been reported by several authors that there may be an OTA 33-B1 intra-articular fracture of the distal femur involving the lateral femoral condyle following a dislocated patella (1). A review of the current literature on reported cases involving LFC osteochondral fractures related to OTA 33-B1 injury due to acute patellar dislocations, as listed in Table 1, showed the following etiologies: fall from standing with rotation of the knee, trauma to the knee when extending a knee in a striking motion or delivering an impact, and trauma related to falling while playing soccer.

Osteochondral Fracture(OCF). Patellar OCF can occur in an adolescent with first-time and also rarely recurrent patellar dislocation in either the patella or on the lateral femoral condyle(OTA 33-B1 fracture site) (2). Therefore, when assessing patients who may have lateral patellar malalignment, careful palpation of the lateral femoral condyle, especially when the knee is flexed, is recommended. The most typical form of an acute lateral dislocation occurs if you slightly bend your knee joint (about 20 degrees-30 degrees), then the patella is in contact with the front lateral rim of the trochlear groove resulting in a typical pattern of injury including the medial inferopatellar region and the avascular non-weightbearing lateral aspect of the front lateral femoral condyle associated with OTA 33-B1 fracture during patellar dislocations (7). The mechanism of these OCDs is impaction of the patella on the lateral femoral condyle trochlear sulcus in a slightly bent position, generating a crushing force, or shear force (5). Fracture usually occurs at the anterior superior margin of the lateral femoral condyle (non-load bearing area), which is the most common site of these OTA 33-B1-type fractures, distinct from the weight-bearing surfaces of the lateral compartment.

According to studies, injuries to the lateral femoral condyle manifested as OTA 33-B1 intra-articular fractures are rare (10). As we go further into the bend of a bent knee, the lesion can involve areas on the weight-bearing surface of the lateral femoral condyle within the OTA 33-B1 fracture zone. The following type of lesion, which compresses on the load-bearing areas in the condyle requires a high degree of flexion angle at the knee joint. This kind of injury can be clinically diagnosed as crush injuries. However, there are few reports about patients who presented with concomitant damage of both weight-bearing and nonweightbearing regions of the LC caused by OTA 33-B1 comminuted fracture due to abrupt dislocation or subluxation of the patella. The mechanism causing this complicated injury may be mainly attributed to major trauma.

Based on previously known injury mechanisms, as well as considering the location and morphology of the osteochondral lesion and the bone marrow edoema seen in our patient, we propose the following pathogenesis of lateral femoral condyle OCIs within OTA 33-B1 intra-articular fracture. Patellar dislocation occurred and at the time of impact the medial side of the patella was in contact with the ground which resulted in high loading of the unloading ramp on the lateral femoral condyle. leading to a breakage at this non-weight-bearing area. The patellar compression was increased and included the weight-bearing area with increasing flexion: ultimately resulting in the development of an OTA 33-B1-related fracture (7).There are other explanations which suggest that shearing stresses, occurring as a result of valgus rotation at the point where the articular surfaces of the tibiofemoral articulation meet, can lead to injury in the mid-lateral part of the lateral femoral condyle (12). In our case, additional mechanical loading by the tibial plateau may have contributed to producing axial compressive forces leading to the OTA 33-B1 intra-articular fracture-associated injury.

Several anatomical risk factors predispose individuals to patellar dislocation and associated osteochondral fractures. Trochlear dysplasia has been widely recognized as a major predisposing factor for patellar instability and has been shown to increase the risk of osteochondral lesions during the dislocation event (13). Similarly, an elevated tibial tubercle–posterior cruciate ligament (TT-PCL) distance, or the more commonly reported tibial tubercle–trochlear groove (TT-TG) distance, reflects excessive lateralization of the tibial tubercle and results in a greater lateral force vector acting on the patella. Previous studies have indicated that both trochlear dysplasia and increased TT-PCL/TT-TG are associated with a higher incidence of lateral femoral condyle osteochondral injuries (2), including OTA 33-B1 type fractures. In the present case, such predisposing anatomical factors may have contributed to the complex fracture pattern involving both weight-bearing and non-weight-bearing areas of the lateral femoral condyle (12).

The weight-bearing zone (14). The treatment of fractures related to OTA 33-B1 intra-articular fracture related to patellar dislocation varies depending on the type of injuries, surgical skills and patient-related variables. Treatment choices include stabilising dislodged osteochondral pieces, removal of mobile bodies, mosaicplasty and chondrocyte implantation (5). Some authors have proposed that any inclusion of the articular surfaces in such fractures of OTA 33-B1 pattern should be reduced surgically and fixed if possible, as untreated lesions are a risk for development of posttraumatic osteoarthritis (15). However, considerable cell death occurs in the dislodged osteochondral fragment, which could be detrimental to the results of fixation (16). The type of fixation chosen (e.g., nonprominent headed compression screw, absorbable implant, sutures) is based on the size of the fragment as well as the quality of the underlying bone of the OTA 33-B1 fracture site (11). If there is not sufficient bone to anchor the fragment, it may also be treated with debridement and microfracture, or other forms of articular surface repair (17).

The arthroscopic debridement in weightbearing and non-weightbearing areas of the lateral femoral condyle showed large fragments and several articular cartilage defects related to the OTA 33-B1 intra-articular fracture of the distal femur. For this reason, an open reduction and internal fixation which led to good clinical outcome in the absence of complications (16). Nevertheless, on the last follow-up, the postoperative MR showed an OTA 33-B1 intra-articular fracture of the distal femur of the lateral femoral condyle, probably related to the primary chondral defect at that site (13). The patient has good walking function, and he does not have any pain with exercise of the knee joint. The remaining cartilage defects, especially at load-bearing regions, can require further treatment later on (17).

Studies have shown that more than 9 out of every 10 lateral dislocations are associated with an MPFL injury (18). As shown in previous studies, cartilage reconstruction procedures require stabilisation of the PFJ to be addressed, as continued instability greatly predisposes to development of OA of this joint (5). There is a wide range of treatment options for first time patellar dislocations with both non-operative as well as surgical treatments available (19). Surgical management has been associated with fewer recurrent episodes of patellar dislocation: non-operative management is still considered as a first-line treatment option (20). However, in those with a high risk of reoccurrence, operative intervention may be an ideal treatment option (21). Many orthopaedic surgeons perform different techniques that target the medial soft tissue injury, such as MPFL reconstruction, medialization procedures, tightrope surgeries, double-barrelled repairs, augmentation techniques and lengthenings (4).

The anatomy of MPFL is well studied and it enables us to perform accurate surgeries (22). Reconstructing MPFL has been shown to be a successful treatment, showing good results for recurrence prevention as well as joint function restoration regardless of used technique or graft type (23).Several studies have demonstrated similar clinical results with respect to recurrence of patellar dislocations using either a primary repair or reconstruction of the MPFL at 2-year postoperative interval (24). Although ligament reconstruction might have better short- and midterm functional outcomes, as well as fewer reoperations, MPFL repair with the addition of synthetic reinforcement can be an alternative to restore patellar stability without the risk of donor site morbidity that comes from harvesting an autograft (8). Lateral retinacular release (LRR): LRR is an adjunctive procedure intended to improve patellar alignment in the trochlear groove by releasing abnormal tight lateral ligaments causing malalignment of the patella (23).

The functional results after surgical treatment of patellar dislocation are generally favorable and not poor (11). However, outcomes are significantly compromised when the patient has concomitant cartilage abrasion or an osteochondral fracture, as the articular surface damage predisposes to posttraumatic osteoarthritis and inferior functional recovery (16). This reinforces the importance of identifying and treating OTA 33-B1 osteochondral lesions in the setting of patellar dislocation. The combination of MPFL reconstruction and lateral retinacular release has been reported as successful by several authors (4).After surgery, the patient had excellent functional recovery and there was no sign of instability with a recurrence of dislocation. Follow-up MRIs showed full healing of the reconstructed MPFL (8).

## Conclusion

Our case as well as other literature suggest that the presence of OTA 33-B1 intra-articular fracture of the distal femur occurring in a weight bearing or non-weight bearing compartment after first time patella dislocation is rare. Our patient underwent MPFL reconstruction with LRR, compared with the regular MPFL reconstruction methods. The patient had good postoperative function and MRI showed the results at the last follow-up. However, clinician must be aware that there is a risk for further damage to the articular cartilage in weight bearing areas associated with this OTA 33-B1 fracture, and therefore may require other treatment interventions if symptoms arise later in life.

## Data Availability

The original contributions presented in the study are included in the article/Supplementary Material, further inquiries can be directed to the corresponding authors.
